# Supplementary material for: Improved Atrial Differentiation of Human Pluripotent Stem Cells by Activation of Retinoic Acid Receptor Alpha (RARα)
Source: J Pers Med. 2022 Apr 13;12(4):628. doi: 10.3390/jpm12040628 (PMC9032956; doi:10.3390/jpm12040628)
Supplement: Supplementary file 1 [file jpm-12-00628-s001.zip › Supplementary_JPM_final.pdf]

Supplemental material

- Figure S1
- Figure S2
- Figure S3
- Supplemental experimental methods
- Table S1-S15
- Supplemental video

Supplemental figures

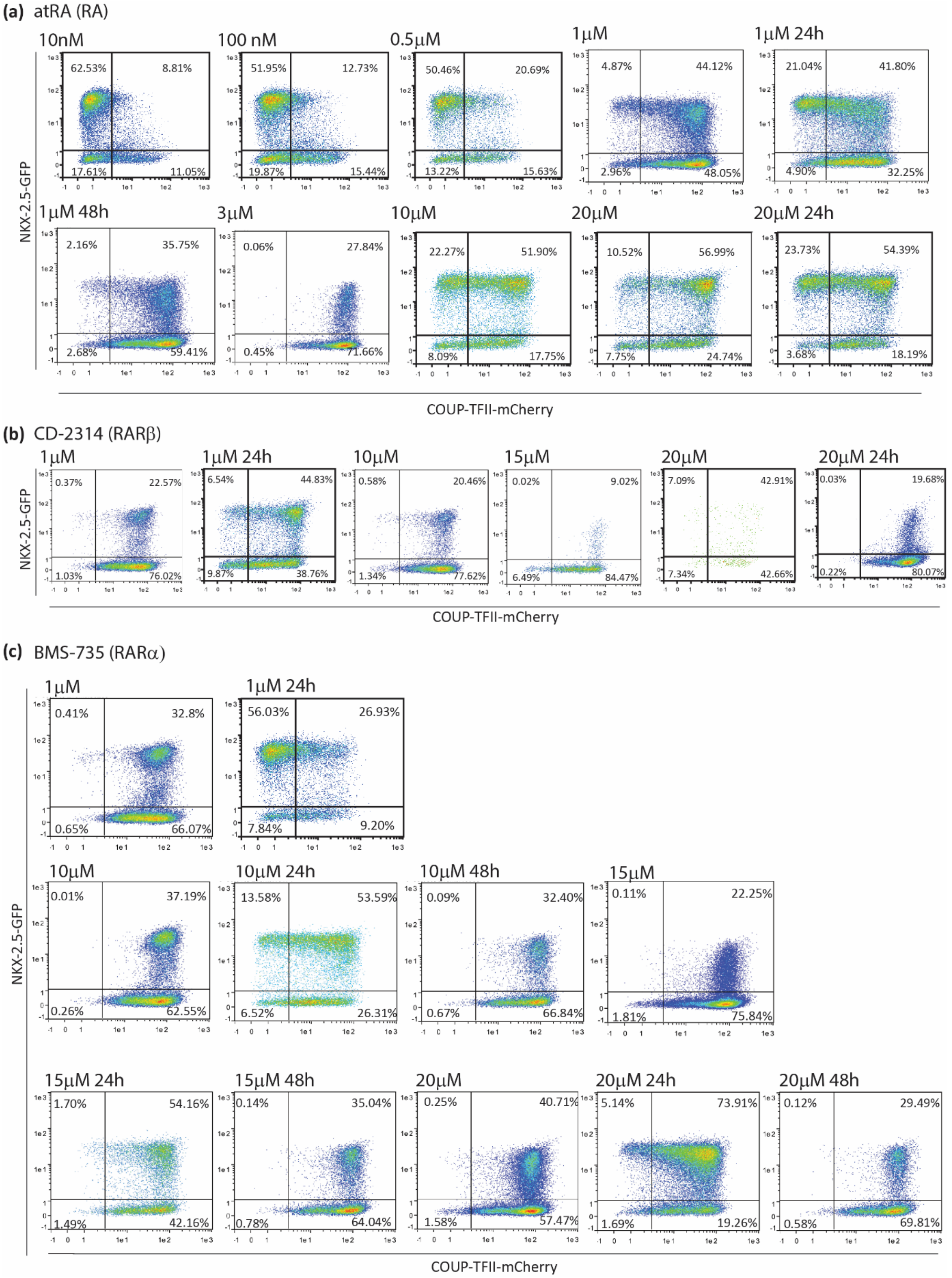

Supplementary Figure S1. Optimization of atrial differentiation with atRA, CD2314 and BMS753. (a-c) Representative flow cytometry plots depicting the percentage of NKX2.5-GFP or COUP-TFII-mCherry-positive cells at day 14 in AM atRA- (a), RARβ- (b) or RARα- (c) induced protocol in spin-EB format. Unless stated, compound was removed a day 7 of differentiation.

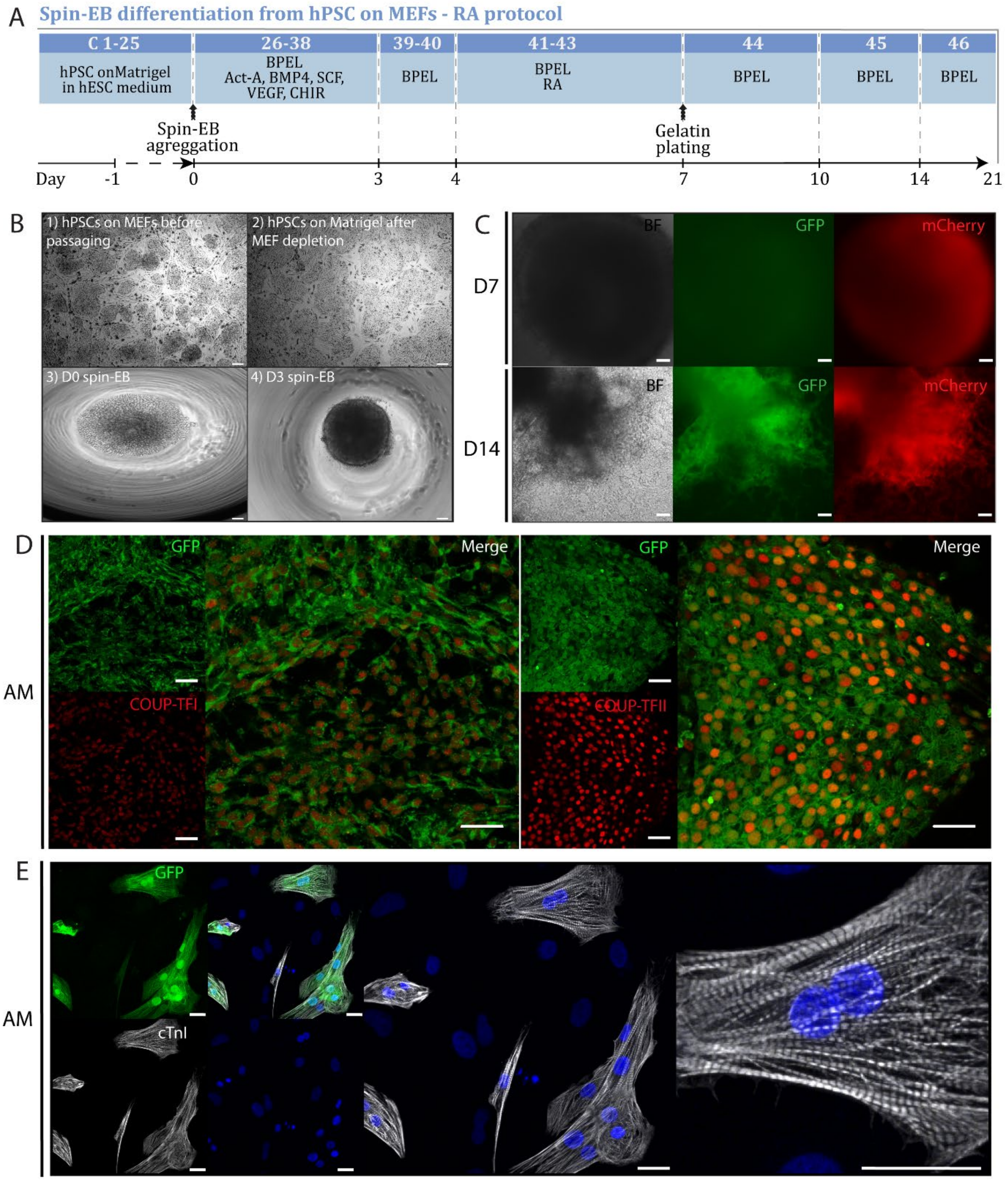

Supplementary Figure S2: Spin-EB differentiation from hPSCs on MEFs with atRA protocol. (a) Schematic representation of the spin-EB from hPSCs grown on irradiated mouse-embryonic feeder (MEF) supported cells. Numbers correspond to step-by-step procedure section B from supplementary materials and methods. (b) 1) HPSCs grown on MEFs in hPSC medium just before passaging, 2) hPSCs after MEF depletion just before EB for-mation, 3) spin-EB just after formation by centrifugation and 4) spin-EB at day 3 of differentiation after cardiac mesoderm induction; scale bar = 100 µm. (c) Spin-EBs from hPSCs grown on MEFs at day 7 and day 14 of differentiation. Endogenous COUP-TFII-mCherry expression together with endogenous NKX2.5-GFP and bright-field (BF) images in AM differentiations. Scale bar = 100 µm. d) Representative pictures of im-munostainings for COUP-TFI (left panel) and COUP-TFII (right panel) together with endogenous NKX2.5-GFP in RA-treated AMs in unsorted cultures after dissociation and re- plating; scale bar = 25 µm, 63x. (e) Representative pictures of immunostainings for cardiac troponin I (cTnI) together with endogenous GFP and nuclear stain DAPI of atRA-treated AMs in unsorted cultures after dissociation and re-plating; scale bar = 25 µm, 63x.

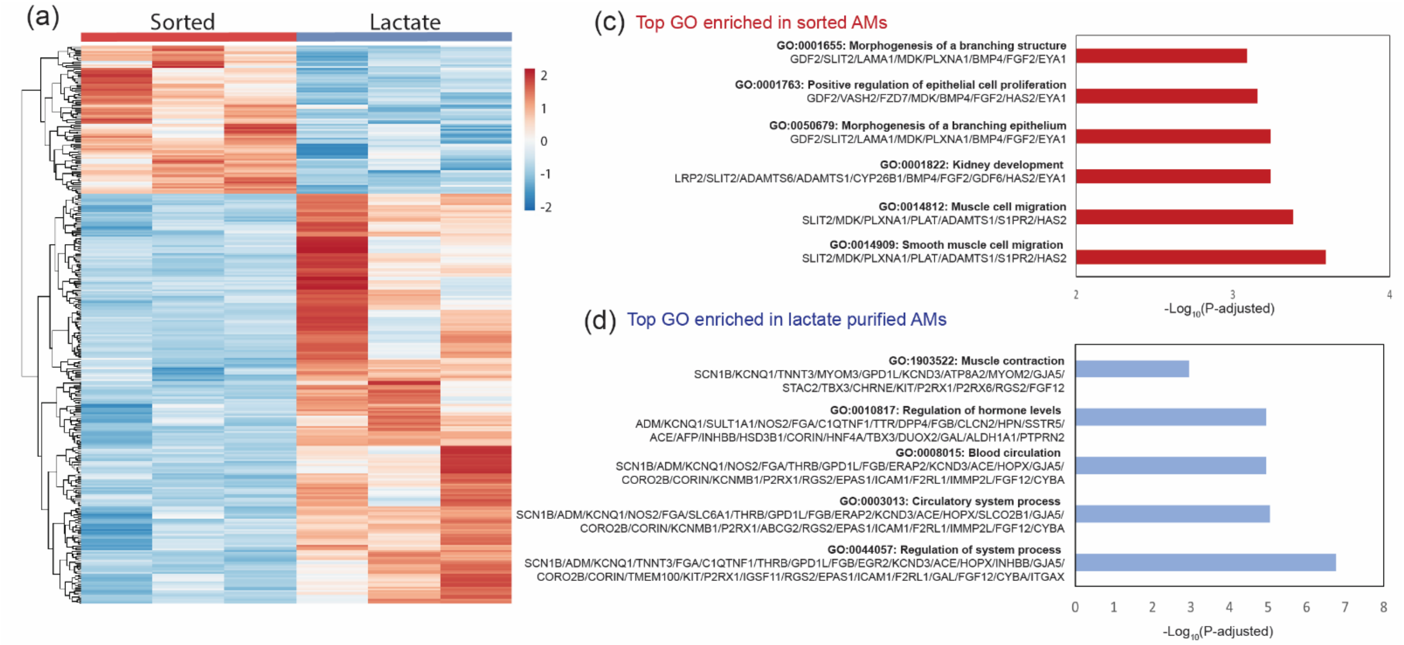

Supplementary Figure S 3: RNA sequencing of RARα-induced atrial cardiomyocytes either sorted or enriched by lactate purification. (a) Heatmap representation of the differentially expressed genes in sorted vs lactate purified AMs (DEGs filtered on log2 fold change 1 and False discovery rate (FDR)-corrected P value < 0.05). (c-d) Top Gene Ontology terms from the upregulated genes in sorted vs lactate AMs (c) or lactate vs sorted AMs (d) AMs.

Supplemental methods

Procedure with step-by-step protocol

A. Spin-EB-based differentiation of AMs from hPSCs grown in defined cultures with E8 and vitronectin

a) Passaging and cryopreservation of hPSCs with EDTA

1) Make sure the E8 culture media (Table S2) is prewarmed and prepare one 15 ml FALCON tube with 4 ml E8 and another tube with 1 ml.
2) Aspirate Vitronectin from the coated plate (Table S14).
3) Add E8 medium (2 ml/well).
4) Take cells from the liquid nitrogen storage vessel and transport on dry ice.
5) Thaw the vial by submerging the vial in the water bath, carefully moving around until there is a pea-sized ice crystal left.
6) Take the vial to the culture hood and spray with ethanol to sterilize the vial.
7) Carefully (cells are very fragile) add 1 ml of the E8 media dropwise to the cells.
8) Transfer the cells into the tube with 4 ml of the E8 media.
9) Wash the vial with 1 ml E8 from the second tube to get the leftover cells.
10) Centrifuge the cell suspension at 240 g for 3 minutes.
11) Resuspend the cells in 500 μl E8.
12) Add the cells to the well previously filled with E8 medium.
13) Wash the tube by adding 500 μl to the tube and add it to the well.
14) Refresh the medium the next day to get rid of any dead cells.
15) Refresh daily with 2 ml E8 medium or excess of medium if hPSCs cannot be refreshed daily, e.g. 8 ml on Friday afternoon for the next refreshment on Monday morning.

For maintaining hPSCs, cells are passaged twice a week (typically Monday or Thursday).

16) Leave E8 at room temperature.
17) Aspirate the medium from the cells and wash with 1 ml DPBS^{-} (Gibco, cat. no. 14190-094).
18) Aspirate the DPBS^{-} and add 1 ml of 0.5 mM EDTA (Invitrogen, cat no. 15575-038) to each well of a 6-well plate with hPSCs.
19) Incubate the cells in EDTA for 3-5 min at 37°C. Note that timing of the EDTA incubation is critical. HPSCs should detach easily, but should not be washed away when removing the EDTA. If incubation was too long, EDTA containing the hPSCs should be collected into a tube for cell collection via centrifugation at 240 g for 3 min before resuspension in E8 medium.
20) Aspirate the EDTA with a 1 ml pipet.
21) Add 1 ml of E8 medium into well of a 6-well plate followed by gentle pipetting up-and-down with a P1000 pipette.
22) Wash all wells with 1 ml E8 and add to the tube with cells.
23) Count the hPSCs with Trypan blue.
24) Seed the hESCs carefully on vitronectin coated plates.
    Typical seeding densities for the COUP-TFII^{mCherry}-NKX2-5^{eGFP} hESC line:
    Split ratio on Monday: 140 K and 160 K and split ratio on Thursday: 80 K and 100 K per well of a 6-well plate. Cell seeding density can be different depending on the passage number of the cells.
25) Refresh daily with 2 ml E8 medium or excess of medium if hPSCs cannot be refreshed daily, e.g. 8 ml on Friday afternoon for the next refreshment on Monday morning.

26) For cryopreservation, hPSCs are resuspended in cold E8 cryopreservation medium (Table S3), transferred to cryovials and subsequently frozen to -80°C in a rate-controlled manner (-1°C/minute). About 24 hours later, the hPSCs are stored in liquid nitrogen at approximately -196˚C.

b) *Formation of EBs from hPSCs grown in E8 on vitronectin*

27) Leave E8 at room temperature.
28) Calculate the number of EBs needed and prepare the number of V-shaped 96 well plates (Table S16) accordingly. In each 96 well plate, 60 wells can be used for making spin EBs. The outer ring of wells should be filled with 150 µl DPBS⁻.
29) Aspirate the medium from the cells and wash with 1 ml DPBS⁻.
30) Aspirate the DPBS⁻ and add 1 ml of 0.5mM EDTA to each well of a 6-well plate with hPSCs.
31) Incubate the cells in EDTA for 3-5 min at 37°C. Note that timing of the EDTA incubation is critical. HPSCs should detach easily, but should not be washed away when removing the EDTA. If incubation was too long, EDTA containing the hPSCs should be collected into a tube for cell collection via centrifugation at 240 g for 3 min before resuspension in E8 medium.
32) Aspirate the EDTA with a 1 ml pipet.
33) Add 1 ml of E8 medium into well of a 6-well plate followed by gentle pipetting up-and-down with a P1000 pipette.
34) Wash all wells with 1 ml E8, add to the cells and count the hPSCs with Trypan Blue.
35) Calculate the volume of cells required to achieve the necessary density of 5K per 50 µl. Calculate 3.5 ml per 96-well plate. Add the necessary components of the EB formation medium (Table S4) to the cell suspension.
36) Seed the hESCs carefully at 5 K per well in 50 µl per 96-well with a multichannel pipette.
37) (Optional) Spin plates at 260 g for 3 min and check EBs under microscope. EBs will also aggregate by gravity if step is omitted.
38) Incubate the cells in the incubator at 37°C with 5% CO_2 for about 24 h. For troubleshooting, see Table 1.

c) *Cardiac mesoderm induction*

39) Prepare the cardiac mesoderm induction medium as indicated in Table S6.
40) Wash the newly formed hPSC EBs with 50 µl per well of plain BPEL and aspirate the BPEL with the multichannel pipette. Note: aspirate all plain BPEL to prevent dilution of the cardiac mesoderm induction medium and ensure correct growth factor concentrations.
41) Add 50 µl of cardiac mesoderm induction medium to each well.
42) Incubate the cells in the incubator at 37°C with 5% CO_2 for about 72 h.
43) Refresh EBs with 50 µl of plain BPEL.
    Note: for generation of ventricular EBs, refresh with 100 µl of plain BPEL instead. Then continue as indicated in point 49.
44) Continue incubation at 37°C with 5% CO_2 for 24 h.

d) *Induction of an atrial phenotype by treatment with Retinoic acid (RA)*

For RA atrial protocol:

45) At day 4, remove plain BPEL and refresh with 100 µl Atrial induction medium for EB differentiation I (Table S7) to direct differentiation towards AMs. Note: aspirate all BPEL to prevent dilution of the RA and ensure proper concentration of RA. For troubleshooting see Table 1.
46) Continue incubation at 37°C with 5% CO_2 until day 7.

For RARα atrial protocol:

45) At day 4, remove plain BPEL and refresh with 50 µl Atrial induction medium for EB differentiation II (Table S8) to direct differentiation towards AMs. Note: aspirate all BPEL to prevent dilution of the BMS and ensure proper concentration of BMS. For troubleshooting see Table 1.
46) Continue incubation at 37°C with 5% CO_2 until day 5.
47) Remove Atrial induction medium II for EB differentiation and add 50 µl of plain BPEL. Removal of BMS at day 5 is critical.
48) Continue incubation at 37°C with 5% CO_2 until day 7.

e) Plating of EBs

49) Coat desired amount of plates with 0.1% gelatin as indicated in Table S14.
50) At day 7 of differentiation, plate EBs on 0.1% gelatin coated plates in 3 ml of plain BPEL medium with a plating density of 30 EBs per well of a 6-well plate. Handle EBs carefully after plating and leave undisturbed for attachment. Optionally, skip plating step and refresh individual EBs in 96-well plate with 100 µl plain BPEL. For troubleshooting, see Table 1.
51) At day 10 of differentiation, refresh plated EBs with 3.5 ml of plain BPEL and place back in incubator.
52) At day 14 of differentiation, refresh plated EBs with 3.5 ml of plain BPEL and place back in incubator. EBs should start to contract between day 10 and 14. For troubleshooting, see Table 1.
53) Dissociate EBs between day 14 and 21 of differentiation for further characterization or downstream application.

B. Spin-EB-based differentiation of AMs from hPSCs grown on MEF feeders

a) Passaging and cryopreservation of hPSCs with TrypLE

1) Seed MEF cells in MEF medium (Table S10) into the required amount of wells of a 6-well plate at a density of 300 – 400K per well 1 till 4 days before seeding the hPSCs.
2) At the day of cell recovery from the liquid nitrogen, remove MEF medium from the well and add 2 mL of fresh hPSC medium (Table S11).
3) Warm up 1 ml and 5 ml hPSC medium in separate 15 ml FALCON tubes.
4) Pick cells from the liquid nitrogen storage vessel and transport on dry ice.
5) Thaw the vial by submerging the vial in the water bath, carefully moving around until there is a pea-sized ice crystal left.
6) Carefully (cells are very fragile) add 1 ml hPSC medium and transfer cells to tube containing the remaining 4 ml hPSC medium.
7) Wash vial with the other 1 ml warm hPSC medium.
8) Spin down for 3 min at 240 g.
9) Take off supernatant and resuspend the cells in 1 mL of hPSC medium.
10) Plate cells on MEF supporter cells.
11) Refresh daily with 3.5 ml hPSC medium or excess of medium if hPSCs cannot be refreshed daily, e.g. 8 ml on Friday afternoon for the next refreshment on Monday morning.

For maintaining hPSCs, cells are passaged twice a week (typically Monday or Thursday).

12) Seed MEF cells into the required number of wells of a 6-well plate at a density of 300 – 400K per well 1 till 4 days before seeding the hPSCs.
13) At the day of passaging remove MEF medium from the well and add 3 mL of fresh hPSC medium.

14) Remove hPSC medium from the cells and wash cells with DPBS-.
15) Add 0.5 mL of 1x TrypLE (Gibco, cat. no. 12563029) and incubate for 3-5 minutes at 37°C. Note: Timing of the TrypLE incubation is critical. HPSCs should detach easily, but should not be incubated too long.
16) Tick to plate to detach cells and pipette gently up and down with a p1000.
17) Transfer cells to a 15 mL FALCON tube already containing 4 mL of MEF medium.
18) Wash the well with an additional 1 ml of medium and add to the cells.
19) Spin down for 3 minutes at 240 g.
20) Take off supernatant and resuspend the cells in 1mL of hPSC medium.
21) Count the cells and calculate the amount of hPSCs to be plated onto the new plate containing MEF supporter cells and hPSC medium.
    Typical seeding densities for the COUP-TFII^{mCherry}-NKX2-5^{eGFP} hESC line:
    Split ratio on Monday: 160 K and 180 K and split ratio on Thursday: 120 K and 140 K per well of a 6-well plate.
22) Refresh daily with 3.5 ml hPSC medium or excess of medium if hPSCs cannot be refreshed daily, e.g. 8 ml on Friday afternoon for the next refreshment on Monday morning.
23) For cryopreservation, hPSCs are resuspended in cold freezing medium (Table S12), transferred to cryovials and subsequently frozen to -80°C in a rate-controlled manner (-1°C/minute). About 24 hours later, the hPSCs are stored in liquid nitrogen at approximately -196˚C).

b) *Formation of EBs from hPSCs grown in hPSC medium on MEFs and cardiac mesoderm induction*

24) Leave hPSC medium at room temperature.
25) HESCs are plated on Matrigel-coated plates (Table S14) in hPSC medium one day before differentiation for feeder depletion at a seeding density of 700 – 800 K per well of a 6-well plate).
26) Warm up the TrypLE, MEF medium and leave BPEL at room temperature.
27) Calculate the number of EBs needed and prepare the number of V-shaped 96 well plates accordingly. In each 96 well plate, 60 wells can be used for making spin-EBs. The outer ring of wells needs to be filled with 150 µl DPBS-.
28) Aspirate the medium from the cells and wash with 2-3 ml DPBS-.
29) Aspirate the DPBS out and add 0.5 ml of warm 1x TrypLE to each well.
30) Incubate the cells in TrypLE for 2-4 min at 37°C, followed by gentle pipetting up-and-down with a P1000.
31) Transfer hPSCs to a 15 ml FALCON tube and add 4.0 ml of MEF medium for each ml of TrypLE.
32) Spin down the cells at 240 g for 3 min. Aspirate the medium and resuspend the cells in 1 ml of BPEL to wash. Important: the wash step with BPEL is critical for the success of differentiation.
33) Spin down cells again at same speed and time. Aspirate the medium and resuspend the cells in 1 ml of BPEL (for every well of a 6-well plate used).
34) Count the cells with Trypan blue and deduct number of MEF supporter cells seeded on Matrigel the day before differentiation.
35) Calculate the amount of cardiac mesoderm induction medium (Table S6) needed and prepare medium with hPSCs. For each well of a 96-well plate, 5 K hPSCs are seeded in 50 µl of cardiac mesoderm induction medium for EB differentiation. Calculate some excess.
36) Add 50 µl of cardiac mesoderm induction medium containing hPSCs to each well using a multichannel pipette. Ensure that the suspension in the reservoir is homogenous by gentle mixing in between.
37) (Optional) Spin plates at 260 g for 3 min and check EBs under microscope.
38) Incubate the cells in the incubator at 37°C with 5% CO_2 for about 72 h. For troubleshooting see Table 1.
39) Refresh EBs with 50 µl of plain BPEL.

Note: for generation of ventricular EBs, refresh with 100 µl of plain BPEL instead. Then continue as indicated in point 45.

40) Continue incubation at 37°C with 5% CO_2 for 24 h.

c) *Induction of an atrial phenotype by treatment with Retinoic acid (RA)*

41) At day 4, remove plain BPEL and refresh with 100 µl Atrial induction medium for EB
42) Differentiation (Table S7). Aspirate all BPEL to prevent dilution of the RA and ensure proper concentration of RA. For troubleshooting see Table 1.
43) Continue incubation at 37°C with 5% CO_2 until day 7.

d) *Plating of EBs*

44) At day 7 of differentiation, plate EBs on 0.1% gelatin coated plates (Table S14) in 3 ml of plain BPEL medium at a plating density of 30 EBs per well of a 6-well plate. Handle EBs carefully after plating and leave undisturbed for attachment. Optional: Skip plating step and refresh individual EBs in 96-well plate with 100 µl plain BPEL. For troubleshooting see Table 1.
45) At day 10 of differentiation, refresh plated EBs with 3.5 ml of plain BPEL and place back in incubator.
46) At day 14 of differentiation, refresh plated EBs with 3.5 ml of plain BPEL and place back in incubator. EBs should start to contract between day 10 and 14. For troubleshooting see Table 1.
47) Dissociate EBs between day 14 and 21 of differentiation for further characterization or downstream application.

C. Dissociation of cardiac differentiations for further characterization

1) Aspirate BPEL medium from the cells and wash with 1 ml DPBS$^-$.
2) Add 700 µl per well (EBs should be covered) of 10X TryplE (Gibco, cat. no. A1217701) and place plate in incubator for 5-10 min. Depending on the day of differentiation, incubation time may depend. For EBs between day 14 and 21, do a first incubation of 8-10 minutes.
3) Tap plate to detach cells and carefully dissociate by pipetting up and down with a p1000 biosphere pipette tip (Table S16). CMs are sensitive to mechanical dissociation, it is recommended not to pipette too harsh. For troubleshooting see Table 1.
4) Place plate back in the incubator for a few minutes and pipette up and down again to dissociate remaining clumps. For EBs between day 14 and 21, 2 to 5 minutes of second incubation may be needed.
5) Add 4 ml BPEL medium in a 15 ml FALCON tube and add the cells to dilute TrypLE.
6) Wash the wells with additional BPEL and transfer to the tube with cells.
7) Spin down at 240 g for 3 min.
8) Optional, but required for cell sorting, filter with filter tube (Table S16).
9) A) Resuspend in Sorting buffer for cell sorting or flow cytometry.
   B) Resuspend in Cardiomyocyte medium plus TDI (Table S12) to plate CMs on vitronectin-coated cell culture plates (Table S14), glass bottom plates (Table S16) or glass coverslips, depending on downstream process.

D. Enrichment of AMs based on metabolic selection

1) On day 13, dissociate the AMs as indicated in section C, and resuspend them in Cardiomyocyte medium plus TDI (Table S12).

2) Seed 1x10[6] AMs on vitronectin-coated cell culture plates (Table S14) with 3 ml per well of Cardiomyocyte medium plus TDI.
3) After 24 h, aspirate Cardiomyocyte medium plus TDI from the cells and wash once with DPBS.
4) Aspirate the DPBS- from the wells and add 3 ml per well of Cardiomyocyte medium for lactate purification plus TDI (Table S13).
5) On day 17, aspirate the medium and add 3 ml of Cardiomyocyte medium plus TDI (Table S12). For troubleshooting see Table 1.
6) Refresh every 3 days until dissociation for characterization or downstream application.

E. Flow cytometry analysis or fluorescence-activated cell sorting (FACS)

1) Perform analysis with a flow cytometer to quantify the percentage of AMs or sort AMs by FACS. For HES3 COUP-red (NKX2.5$^{eGFP/wt}$-COUP-TFII$^{mCherry/wt}$) hESC, AMs are selected by NKX-2.5-GFP+ and COUP-TFII-mCherry+ expression and as control, VMs are selected by NKX-2.5-GFP+ and COUP-TFII-mCherry- expression. For LUMC0020iCTRL-06 hiPSCs SIRPA+/VCAM- expression can be used to sort for AMs and SIRPA+/VCAM+ can be used to sort for VMs.
For flow cytometric analysis, we use the flow cytometer MACSQuant VYB from Miltenyi Biotec; for cell sorting, we use the SH800S cell sorter from Sony Biotechnology. In our lab, FlowJo software is used for data analysis.
2) Resuspend in Cardiomyocyte medium plus TDI to plate sorted AMs on vitronectin-coated cell culture plates, glass bottom plates or glass coverslips depending on downstream process.

F. Immunotyping of hPSC-derived AMs

10) Fixate cells in 2% Paraformaldehyde (PFA, Merck, cat. no. 1.04005.1000) for 20 min at room temperature.
11) Wash 3x in DPBS-. Fixed cells can be stored at 4°C in DPBS-.
12) Permeabilize in 0.1% Triton X-100 (Sigma-Aldrich, cat. no. T8787) for exactly 8 min. Permeabilization time is critical.
13) Wash 3x in DPBS-.
14) Block in 5% Fetal Bovine Serum (FBS, Sigma Aldrich, cat no. F7524) for 1 h at room temperature.
15) Incubate with primary antibody overnight at 4°C in 5% FBS:
    - Mouse anti-COUP-TFI (H8132, Perseus Proteo, 1:500)
    - Mouse anti-COUP-TFII (H7147, Perseus Proteomics, 1:500)
    - Mouse anti-cTnI (clone H-170, Santa Cruz, 1:500)
    - Mouse-anti-cTnT (clone 13-11, ThermoFisher MA512960, 1:400)
    - Rabbit anti-NKX2.5 (clone E1Y8H, Cell Signaling, 1:400)
16) Wash 3x 5 min in DPBS-.
17) Incubate with secondary antibody for 90 min at room temperature in 5% FBS.
    - Goat-anti Rabbit or anti Mouse Cy3 (Jackson Immunoresearch, 1:250).
    - Goat-anti Rabbit Alexa Fluor 4.88 (Invitrogen, A27034, 1:400).
18) Wash 3x 5 min in DPBS-.
19) Wash shortly in DI water.
20) Mount in Prolong-Gold with DAPI (Thermo Fisher Scientific cat no. P36935) or perform DAPI staining separately before, and dry slides overnight at room temperature. Slides can be stored at room temperature in the dark before imaging.

G. Membrane potential characterization of hPSC-derived AMs with FluoVolt™ Kit

Prepare solutions according to the manufacturer of the FluoVolt™ membrane potential kit (Themo Fisher Scientific cat no. F10488):

1) Prepare a fresh FluoVolt™ Loading solution by mixing PowerLoad™ concentrate (Component B, 1:100) and FluoVolt™ dye (Component A, 1:1000). Vortex to mix. Add the required volume of CM plus TDI medium. Invert to mix and protect from light.
2) Remove the medium from the adherent cells plated on Vitronectin-coated cell culture plates and wash twice with CM plus TDI medium.
3) Add 50 µL (for a well of a 96-wells plate) of FluoVolt™ Loading solution (from step 1) to the cells. Incubate cells at room temperature in the dark for 30 minutes.
4) Remove FluoVolt™ Loading Solution and wash cells twice in CM plus TDI medium.
5) Add 100 µL (for a well of a 96-wells plate) of CM plus TDI medium.
6) Add 1:10 diluted Neuro Backdrop Background Suppressor solution (Component C) to suppress background fluorescence (Optional).
7) Image cells loaded with FluoVolt™ dye according to manufacturer's protocol.

H. Quantitative real-time PCR

1. Lysate AMs or VMs isolate RNA with the NucleoSpin RNA isolation Kit (Macherey-Nagel) according to the manufacturer's protocol.
2. Quantify RNA with Nanodrop ND-1000 Spectrophotometer or similar.
3. Approximately 500ng cDNA are synthesized with the iScript cDNA Synthesis Kit (Bio- Rad) according to manufacturer's protocol.
4. Perform real-time quantitative PCR (qPCR) in triplicate using the SensiMix SYBR (Meridian) and the CFX384 Real-time PCR detection system. Use 6ng of cDNA per well. Use non-template reactions (RNase-free H2O instead of cDNA) as negative controls. Run qPCR reactions with a 3-Step protocol: 10 min at 95°C, followed by 40 cycles of 95°C for 15s, 60°C for 15s and 72°C for 15s. Primer sequence information is provided in Table S15.
5. Analyze data with Bio-Rad CFX Manager software. RPLP0 is used as housekeeping gene and fold changes in gene expression are calculated relative to ventricular cardiomyocytes.

Supplementary tables

Table S1. Percentages of atrial (AM) and ventricular (VM) myocytes after different treatments

	AM	VM
**1 µM atRA 24 h**	31.8 ± 4.6	44.0 ± 7.3
**1 µM atRA 48 h**	35.8 ± 0.0	2.2 ± 0.0
**3 µM atRA 72 h**	36.6 ± 4.5	21.0 ± 10.5
**10 µM atRA 72 h**	52.9 ± 1.0	23.3 ± 1.0
**20 µM atRA 72 h**	57.7 ± 0.8	8.5 ± 2.0
**20 µM atRA 24 h**	56.1 ± 1.8	21.1 ± 2.7
**1 µM CD 72 h**	30.5 ± 7.2	2.2 ± 1.1
**1 µM CD 24 h**	52.8 ± 4.1	6.2 ± 0.5
**10 µM CD 72 h**	15.0 ± 3.2	0.0 ± 0.0
**15 µM CD 72 h**	9.0 ± 0.0	0.0 ± 0.0
**20 µM CD 72 h**	42.6 ± 0.4	7.4 ± 0.3
**20 µM CD 24 h**	24.3 ± 4.6	0.0 ± 0.0
**1 µM BMS 72 h**	34.7 ± 3.0	8.0 ± 7.6
**1 µM BMS 24 h**	30.2 ± 2.2	53.8 ± 2.1
**10 µM BMS 72 h**	32.8 ± 6.5	0.1 ± 0.0
**10 µM BMS 24 h**	50.6 ± 4.5	12.3 ± 5.7
**10 µM BMS 48 h**	32.4 ± 0.0	0.1 ± 0.0
**15 µM BMS 72 h**	29.0 ± 9.4	0.1 ± 0.0
**15 µM BMS 24 h**	61.2 ± 3.6	7.0 ± 2.7
**15 µM BMS 48 h**	35.0 ± 0.0	0.1 ± 0.0
**20 µM BMS 72 h**	44.5 ± 2.6	0.2 ± 0.1
**20 µM BMS 24 h**	72.3 ± 0.6	5.2 ± 0.7
**20 µM BMS 48 h**	29.5 ± 0.0	0.1 ± 0.0

Table S2. Essential 8 (E8) medium composition

Composition *	Supplier	Final concentration
**Essential 8 basal medium**	Thermo Fisher, cat no. A1517001	-
**Essential 8 supplements medium**	Thermo Fisher, cat no. A1517001	2 %
**Penicillin-Streptomycin (5,000 U/mL)**	Thermo Fisher, cat no. 15070063	1 x

* Medium can be stored for two weeks at 4°C or frozen at -20°C for 1 month.

Table S3. E8 cryopreservation medium

Composition	Supplier	Final concentration
**E8 medium**	As indicated in Table 1	-
**Dimethyl sulfoxide, DMSO**	Sigma Aldrich, cat no. D2650	10 %

**Revita cell supplement optional (for iPSC recommended)**	Thermo Fisher, A2644501	1 x

Table S4. MEF medium

Composition *	Supplier	Final concentration
**DMEM/F12**	Gibco, cat. no. 11320074	10 %
**Fetal Bovine Serum**	Sigma Aldrich, cat no. F7524	0.5 x
**Penicillin-Streptomycin**	Thermo Fisher, cat. no. 15070063	1 x
**Glutamax**	Thermo Fisher, cat no. 35050038	1 x
**Non-essential amino acids**	Gibco, cat. no. 11140050	10 %

* Medium can be stored at 4°C for 2 weeks and at -20°C for up to a year.

Table S5. HPSC medium

Composition *	Supplier	Final concentration
**Knock Out Serum Replacement**	Gibco, cat. no. 10828010	20 %
**2 ME ß-mercaptoethanol**	Thermo Fisher, cat. no. 31350010	0.1 mM
**Penicillin-Streptomycin**	Thermo Fisher, cat. no. 15070063	0.5 x
**Non-essential amino acids**	Gibco, cat. no. 11140050	1 x
**DMEM/F12**	Gibco, cat. no. 11320074	-
**Human FGF-2 IS, premium grade****	Miltenyi Biotec, cat. no. 130-104-923	10 ng/ml

* Medium can be stored at 4°C for 1 week and at -20°C for up to a year, without FGF-2.
** Filter the medium before adding FGF-2.

Table S6. HESC freezing medium

Composition *	Supplier	Final concentration
**Knock Out Serum Replacement**	Gibco, cat. no. 10828010	50 %
**DMEM/F12 with Glutamax**	Thermo Fisher, cat. no. 31331093	35 %
**Ethyleneglycol**	Sigma Aldrich, cat no. 102466-1L-M	10 %
**DMSO**	Sigma Aldrich, cat no. D2650	5 %

* Prepare medium fresh and keep cold on ice before use.

Table S7. EB formation medium

Composition*	Supplier	Final concentration
**E8 medium**	As indicated in Table 1	-
**Poly (vinyl alcohol)**	Sigma Aldrich, cat no. P8136-250G	400 μg/ml
**ROCK Inhibitor, Y-27632**	Bio-Connect, cat no. S1049-5MG	10 μM

* Prepare EB formation medium always fresh before use.

Table S8. BPEL medium, as described in [1]

Composition *,**	Supplier	Final concentration
**IMDM, no phenol red**	Thermo Fisher, cat. no. 21056023	43 %
**Ham's F-12 Nutrient Mix, GlutaMAX™ Supplement**	Thermo Fisher, cat. no. 31765027	43 %
**PFHM-II Protein-Free Hybridoma Medium**	Thermo Fisher, cat. no. 12040077	5 %

**Poly (vinyl alcohol)**	Sigma Aldrich, cat. no. P8136-250G	0.125%
**Phenol Red**	Sigma Aldrich, cat. no. P3532-5G	0.011 g
**Chemically defined lipid concentrate**	Thermo Fisher, cat. no. 11905031	1x
**1-Thioglycerol (α-MTG)**	Sigma Aldrich, cat. no. M6145-25ML	448 μM
**GlutaMAX Supplement**	Thermo Fisher, cat. no. 35050038	1x
**Insulin-Transferrin-Selenium Ethanolamine (ITS-X)**	Thermo Fisher, cat. no. 51500-056	0.1x
**Ascorbic acid-2P**	Sigma Aldrich, cat. no. A8960-5G	50 μg/ml
**Penicillin-Streptomycin**	Thermo Fisher, cat no. 15070063	0.5 x
**BSA**	Bovostar, cat. no. BSAS 0.5	0.25 %

* Sterilize the medium with a stericup filter.
** Medium can be stored up to two weeks at 4°C or up to 6 months at -20°C

Table S9. Cardiac mesoderm induction medium for EB differentiation

Composition *	Supplier	Final concentration	Concentration range **
**BPEL medium**	As indicated in Table 4	-	-
**Activin-A**	Miltenyi Biotec, cat. no. 130-095-547	1.5 μM	20 - 35 ng/μl
**BMP4**	Miltenyi Biotec, cat. no. 130-095-549	30 ng/ml	20 - 35 ng/μl
**CHIR-99021**	Tocris Bioscience, cat. no. 4423	30 ng/ml	-
**SCF**	Stem Cell Technologies, cat no. 78062.1	40 ng/ml	30 - 50 ng/μl
**VEGF**	Miltenyi Biotec, cat no. 130-109-386	30 ng/ml	20 - 40 ng/μl

* Prepare medium fresh before use.
** The concentration of growth factors is critical for the success of differentiation and needs to be optimized for every hPSC line and batch of growth factors.

Table S10. Atrial induction medium for EB differentiation I

Composition *	Supplier	Final concentration
**BPEL medium**	As indicated in Table 4	-
**All-trans RA****	Sigma Aldrich, cat no. R2625	1 μM

* Prepare medium fresh before use, in the dark and protect from light.
** RA is light, heat and air sensitive, handle in the dark on ice and quick.

Table S11. Atrial induction medium for EB differentiation II

Composition *	Supplier	Final concentration
**BPEL medium**	As indicated in Table 4	-
**BMS-735****	Tocris Bioscience, cat.no. 3505	20 μM

* Prepare medium fresh before use, in the dark and protect from light.
** BMS-735 is light, heat and air sensitive, handle in the dark on ice and quick.

Table S12. Cardiomyocyte medium plus TDI, as described in [2]

Composition*,**	Supplier	Final concentration

**Dulbecco's Modified Eagle's Medium**	Sigma Aldrich, cat no. D5030-10x1L	111 mM Na
**D(+)-Glucose**	Millipore, cat no. 1083371000	15 mM
**Sodium 3-hydroxybutyrate**	Sigma Aldrich, cat no. 54965-10G	0.19 mM
**L-Carnitine hydrochloride**	Sigma Aldrich, cat no. C0283-5G	0.5 mM
**Creatine monohydrate**	Sigma Aldrich, cat no. C3630-100G	1 mM
**Taurine**	Sigma Aldrich, cat no. T8691-100G	5 mM
**Phenol Red**	Phenol Red (Sigma Aldrich, cat no. P3532-5G)	31 μM
**DI Water**	Thermo Fisher, cat no. 15230097)	-
**Sodium Pyruvate**	(Thermo Fisher, cat no. 11360039)	0.5 mM
**Trace elements A**	Cellgro, cat no. 25-021-CI	1x
**Trace elements B**	Cellgro, cat no. 25-022-CI	1x
**Trace elements C**	Cellgro, cat no. 25-023-CI)	1x
**Chemically defined lipid concentrate**	Thermo Fisher, cat no. 11905031	1x
**1-Thioglycerol (α-MTG)**	Sigma Aldrich, cat. no. M6145-25ML	448 μM
**GlutaMAX Supplement**	Thermo Fisher, cat. no. 35050038	1x
**Insulin-Transferrin-Selenium Ethanolamine (ITS-X)**	Thermo Fisher, cat no. 51500-056)	0.01x
**Ascorbic acid-2P**	Sigma Aldrich, cat no. A8960-5G	50 μg/ml
**Penicillin-Streptomycin**	Thermo Fisher, cat. no. 15070063	0.5 x
**BSA**	Bovostar, cat. no. BSAS 0.5	0.25 %
**Sodium hydrogen carbonate (NaHCO3)**	Millipore, cat no. 1063290500	3.7 g/L
**3′,5-Triiodo-L-thyronine sodium salt (T3)**	Sigma Aldrich, cat no. T6397-100G	100 nM
**Dexamethasone**	(Tocris, cat no. . 1126/100)	1 μM
**Long R3 IGF-I human (IGF)**	Sigma Aldrich, cat no. I1271	100 ng/ml

* Bring the medium to pH 7.3-7.4 and filter medium with stericup filter.
** Medium can be frozen to -20°C without TDI components, which can be added after thawing and before use. After thawing, medium can be used for up to two weeks at 4°C. With TDI components added, use for up to one week at 4°C.

Table S13. Cardiomyocyte medium for lactate purification plus TDI

Composition*,**	Supplier	Final concentration
**Cardiomyocyte medium without glucose**	Table 12, minus D(+)-Glucose	-
**Sodium DL-lactate solution**	60%, Sigma Aldrich, cat. no. L4263	5 mM

* Bring the medium to pH 7.3-7.4 and filter medium with stericup filter.
** Medium can be frozen to -20°C without TDI and DL-lactate components, which can be added after thawing and before use. After thawing, medium can be used for up to two weeks at 4°C. With TDI components added, use for up to one week at 4°C.

Table S14. Primer sequences for RT-qPCR

Primer	Forward	Reverse
HARP	CACCATTGAAATCCTGAGTGATGT	TGACCAGCCCAAAGGAGAAG
NR2F2	AAGCCATCGTGCTGTTCAC	GCTCCTCACGTACTCCTCCA
PITX2	GTGTGGACCAACCTTACGGA	AGCCATTCTTGCATAGCTCG
ISL1	TTTATTGTCGGAAGACTTGCCACTT	TCAAAGACCACCGTACAACCTTTATCT
GJA5	TCTTTCCCTAACCCGATCC	TGTCCCTGGCCTTGAATATC
KCNJ3	TGTCGTCATCCTAGAAGGCA	AAAAACGATGACCCCAAAGA
MYL2	GATGTTCGCCGCCTTCCCCC	GCAGCGAGCCCCCTCCTAGT
IRX4	TTCCGTTCTGAAGCGTGGTC	TGAAGCAGGCAATTATTGGTGT
